# Revealing the Modulatory Role of Microsporidian circRNAs in the Infection of Honey Bee Workers

**DOI:** 10.3390/insects17050513

**Published:** 2026-05-19

**Authors:** Yaqin Gao, Zhenzhen Zuo, Kaiyao Zhang, Jingxian Li, Genchao Gan, Yuwei Zhang, Shuai Zhou, Jianfeng Qiu, Dafu Chen, Rui Guo

**Affiliations:** 1 College of Bee Science, Fujian Agriculture and Forestry University, Fuzhou 350002, China; 18789833015@163.com (Y.G.); 15064948756@163.com (Z.Z.); kaiyao1223@126.com (K.Z.); 18236228430@163.com (J.L.); 19220444171@163.com (G.G.); 13759839701@163.com (Y.Z.); 15837656300@163.com (S.Z.); jfqiu@fafu.edu.cn (J.Q.); 2 National & Local United Engineering Laboratory of Natural Biotoxin, Fuzhou 350002, China; 3 Apitherapy Research Institute of Fujian Agriculture and Forestry University, Fuzhou 350002, China

**Keywords:** *Vairimorpha ceranae*, *Apis mellifera ligustica*, circRNA, ceRNA, host–pathogen interaction

## Abstract

*Vairimorpha ceranae* (formerly *Nosema ceranae*) is an obligate intracellular parasite that poses a major threat to the health of the honey bee. Circular RNAs (circRNAs) have been recognized as key regulators in gene expression and pathogen–host interactions. However, their expression patterns and regulatory roles in *V. ceranae* infection remain largely unexplored. In this study, we performed circRNA profiling in *V. ceranae* spores (NcCK) and the midguts of *Apis mellifera ligustica* workers at 7 d post inoculation (dpi) and 10 dpi (Nc7T and Nc10T) based on transcriptome sequencing, followed by in-depth investigation of the regulatory roles of differentially expressed circRNAs (DEcircRNAs). In total, 243 circRNAs were identified in *V. ceranae*, with lengths predominantly ranging from 201 to 400 nucleotides. Comparative analysis screened 70 and 192 DEcircRNAs in the NcCK vs. Nc7T and NcCK vs. Nc10T comparison groups, respectively, with a significant majority being downregulated. The parental genes of these DEcircRNAs were significantly enriched in fundamental cellular processes and critical pathways such as protein processing in the endoplasmic reticulum and ribosome biogenesis. Additionally, we constructed a competing endogenous RNA network, suggesting that DEcircRNAs could potentially interact with DEmiRNAs to modulate mRNAs associated with fungal proliferation-relevant signaling pathways like MAPK, PI3K–Akt, and cAMP. Moreover, numerous DEcircRNAs were predicted to contain internal ribosome entry site elements, indicative of their potential for protein coding. The back-splicing junctions and expression trends of selected DEcircRNAs were successfully validated by RT-PCR and qRT-PCR. Our data not only offer a valuable resource for future functional studies but also provide a basis for elucidating the circRNA-mediated mechanisms underlying microsporidian pathogenesis and host–pathogen interactions.

## Simple Summary

The health of *Apis mellifera ligustica* is critically threatened by the microsporidian parasite *Vairimorpha ceranae* (formerly *Nosema ceranae*), which infects the midgut and contributes to worldwide colony losses. Circular RNAs (circRNAs) are a stable and conserved class of non-coding RNAs that play a pivotal part in modulating gene expression and pathogenesis. However, little is known about the roles of circRNAs in *V. ceranae* infection. This study employed deep sequencing and bioinformatics to interpret the regulatory roles of differentially expressed circRNAs (DEcircRNAs) in the *V. ceranae* infection process. A total of 243 circRNAs were identified in the *V. ceranae*. A total of 70 and 192 circRNAs were observed to be differentially expressed in the midguts of worker bees at 7 d and 10 d post inoculation with *V. ceranae*. The parental genes of DEcircRNAs were enriched in vital pathways like protein processing in the endoplasmic reticulum and ribosome biogenesis. The target mRNAs in the DEcircRNA-involved regulatory networks were engaged in several crucial signaling pathways such as MAPK, PI3K–Akt, and cAMP. Additionally, a subset of DEcircRNAs were found to contain internal ribosome entry sites (IRES), suggestive of potential protein-coding capacity. These findings depict the first expression pattern and regulatory network of circRNAs in *V. ceranae* infection and offer epigenetics insights into the pathogenesis of *V. ceranae*.

## 1. Introduction

Honey bees are irreplaceable pollinators in nature, playing a crucial part in maintaining ecological balance as well as ensuring global food security [1]. *Apis mellifera ligustica*, a bee species valued for its excellent production traits, is widely utilized in apiculture, holding significant ecological, research, and economic importance [2]. Microsporidia comprise a phylum of single cell, intracellular parasites and represent the earliest diverging branch in the fungal kingdom [3]. *Vairimorpha ceranae* (formerly *Nosema ceranae*), an obligate parasitic pathogen, is now recognized as a major threat to global honey bee health. Infection by *V*. *ceranae* significantly inhibits the apoptosis of midgut epithelial cells, disrupts intestinal tissue homeostasis, and impairs the host’s defense capability against other stress [4]. Furthermore, a consistent increase in spore load with the proliferation of *V. ceranae*, leading to persistent energetic stress and midgut epithelial cell structural damage to the host, ultimately resulting in a shortened lifespan [5]. As obligate intracellular parasites with drastically reduced genomes, all microsporidia rely heavily on exploiting the host’s molecular machinery for survival and proliferation [6]. However, due to historical technical bottlenecks, such as the difficulty of genetic manipulation caused by the rigid spore wall, elucidating the exact host–pathogen molecular interactions has been remarkably challenging across the entire phylum. Consequently, the lack of broad-spectrum and highly effective treatments remains a significant hurdle for microsporidian infections in general.

Circular RNAs (circRNAs) are a class of non-coding RNA (ncRNA) molecules characterized by a covalently closed circular structure. They are widely expressed in eukaryotes, exhibiting high stability, conservation, and tissue specificity [7]. Increasing evidence has demonstrated that circRNAs are engaged in modulating cellular activities by acting as microRNA sponges, binding to RNA-binding proteins (RBPs) or participating in transcriptional regulation [8]. Although studies on circRNAs in fungi started relatively late, they have become a new frontier. On the basis of high-throughput sequencing technologies, diverse circular RNAs (circRNAs) with distinct functions have been characterized in several important fungal pathogens for humans and plants. For instance, in the human pathogenic fungus *Aspergillus fumigatus*, many circRNAs originate from exonic regions of genes related to stress response and virulence, suggesting their potential in regulating virulence factor expression and involvement in host–pathogen interactions [9]. The presence of a large number of circRNAs has been predicted and validated in *Cryptococcus neoformans*—an opportunistic fungal pathogen of humans that causes cryptococcosis—with their functions potentially involving adaptation to environmental stressors such as host body temperature and oxidative stress [10]. These studies together depict a landscape where circRNAs represent a novel layer in the regulation of fungal virulence and adaptability.

Microsporidia, including *V. ceranae*, possess drastically reduced genomes and lack many canonical eukaryotic metabolic and regulatory pathways, giving rise to highly specialized and unique gene expression regulatory mechanisms [11]. However, the current understanding of circRNAs in microsporidia is still scarce. Hence, investigating the circRNAs produced by both the honey bee host and the pathogen during *V. ceranae* infection, along with their interactive relationships, holds transformative significance for elucidating its pathogenic mechanisms. This study employs small RNA-Seq and bioinformatics to conduct transcriptome-wide identification and differential expression profiles of circRNAs in *V. ceranae* during the infection of *A. m. ligustica* workers, followed by an in-depth investigation of parental genes of differentially expressed circRNAs (DEcircRNAs) as well as their competing endogenous RNA (ceRNA) regulatory networks. Our findings will not only shed light on the mechanisms underlying circRNA-regulated *V. ceranae* infection, but also open the door for developing circRNA-based diagnosis and treatment tools for bee nosemosis.

## 2. Materials and Methods

### 2.1. Biological Materials

The *V. ceranae* spores were purified and maintained by the Bee Protection Laboratory, College of Bee Science and Biomedicine, Fujian Agriculture and Forestry University [12]. Workers of *A. m. ligustica* were collected from the bee colonies maintained by the Bee-Pathogen Interaction Mechanisms research team at the same institution.

### 2.2. Purification of the V. ceranae Strain

Spores of *V. ceranae* were extracted and purified following the method described by Guo et al. [13]. Midguts from infected *A. m. ligustica* foragers were dissected, homogenized, and filtered. The homogenate was centrifuged at 845 rcf for 15 min. The pellet was resuspended in sterile water and centrifuged again at 2348 rcf for 15 min, repeated twice. The spore pellet was then resuspended in 25% Percoll and layered onto a pre-formed Percoll density gradient (100%, 75%, 50%, and 25%). Gradient centrifugation was performed at 12,210 rcf for 30 min. The milky-white spore band was carefully aspirated to obtain purified spores. A portion of these purified spores was rapidly frozen in liquid nitrogen and stored at −80 °C (SANYO, Chūō-ku, Japan) (designated as the control group, NcCK), while the remainder was stored at 4 °C for subsequent inoculation.

### 2.3. Spore Inoculation and Midgut Sample Preparation

Inoculation of *A. m. ligustica* worker bees and the preparation of midgut samples were performed according to established protocols [14]. In brief, the procedure was as follows: strong and apparently healthy colonies were selected. Newly emerged worker bees (0-day-old) were placed in a constant temperature incubator maintained at 34 ± 0.5 °C. These were divided into six plastic boxes at a density of 35 bees per box, with each box provided ad libitum access to 50% (*w*/*v*) sterile sucrose solution for overnight acclimation. After being starved for 2 h, bees in three of the boxes were individually fed 5 μL of sucrose solution containing 1 × 10^6^ *V. ceranae* spores. Midgut samples were collected at 7 days post-inoculation (Nc7T) and 10 days post-inoculation (Nc10T). For each sample, midguts from three bees were pooled into one RNA-free microcentrifuge tube. Three biological replicates were prepared for each group. All samples were immediately flash-frozen in liquid nitrogen and stored at −80 °C until further analysis.

### 2.4. RNA Extraction, cDNA Library Construction, and Deep Sequencing

Total RNA was extracted from *V. ceranae* spores (NcCK) and infected midgut samples (Nc7T, Nc10T) using TRIzol reagent (Transgene S.A., Illkirch-Graffenstaden, France). Ribosomal RNA was removed, and linear RNA was digested with RNase R. The enriched circRNA was fragmented and reverse-transcribed using random hexamers. Double-stranded cDNA was synthesized, purified, and used to construct libraries following standard Illumina protocols. Libraries were sequenced on an Illumina HiSeq^TM^ 2500 platform (Illumina Inc., San Diego, CA, USA).

### 2.5. Quality Control and Filtration of Sequencing Data

Raw sequencing data of NcCK, Nc7T and Nc10T *V. ceranae* were processed to obtain high-quality (HQ) clean reads. The HQ clean reads from each sample were aligned to the *V. ceranae* reference genome (GCF_000182985.1) using TopHat, generating alignment results for each individual sample. Unmapped reads were extracted from these alignment results. Subsequently, both ends (default 20 bp) of each unmapped read were extracted to create ‘anchor reads’. These anchors reads were then realigned to the genome. The resulting alignment files were submitted to the Find-circ software version 1.2 for the identification of circular RNAs.

### 2.6. Source of microRNA Omics Data

The small RNA sequencing (sRNA-Seq) was previously performed on *V. ceranae* spores and on the midguts of *A. m. ligustica* worker bees at 7 days and 10 days post-inoculation with *V. ceranae*, generating high-quality small RNA-Seq data. The relevant data have been deposited in the NCBI SRA database under BioProject accession numbers PRJNA395264 (NcCK) and PRJNA406998 (Nc7T, Nc10T) [15].

### 2.7. Screening of DEcircRNAs and Analysis of Their Parental Genes

The expression level of each circRNA was calculated using the RPM (mapped back-splicing junction reads per million mapped reads) method. The lg(RPM + 1) values of circRNAs in the treatment and control midgut samples were computed. Differentially expressed circRNAs (DEcircRNAs) between the comparison groups (NcCK vs. Nc7T and NcCK vs. Nc10T) were identified using DESeq2 software version 1.0 [16], with the following screening criteria: |log2 (Fold change)| ≥ 1, and *p* ≤ 0.05. CircRNAs can interact with RNA polymerase II, U1 snRNP, and gene promoters to regulate the expression of their parental genes. The anchor reads from both ends of the DEcircRNAs were aligned to the *V. ceranae* reference genome (assembly ASM98816v1) using the short-read aligner Bowtie [17]. A gene was identified as the host gene of a circRNA if both ends aligned to the same gene. These parental genes were then mapped to the GO and KEGG databases to annotate functional terms and KEGG pathways, respectively.

### 2.8. Targeting Relationship Prediction and Regulatory Network Analysis

Based on the previously obtained microRNA (miRNA) omics data, miRNAs potentially targeted by and binding to the DEcircRNAs were screened to establish DEcircRNA–miRNA regulatory relationships. The target mRNAs of these miRNAs were further predicted, and a DEcircRNA–miRNA–mRNA regulatory network was constructed accordingly. The network was visualized using Cytoscape v.3.2.1 software. GO functional and KEGG pathway enrichment analyses were subsequently performed on the identified target mRNAs.

### 2.9. Prediction and Analysis of the Protein-Coding Potential of DEcircRNAs

The identification of putative internal ribosome entry site (IRES) elements within the DEcircRNAs was performed using the IRESfinder software version1.1.0. Subsequently, the ORFfinder tool was employed to predict open reading frames in the DEcircRNA sequences.

### 2.10. PCR Validation

Based on the nucleotide sequences of these circRNAs, divergent primers spanning the back-splicing junction sites were designed using Primer Premier 5 software (Table 1). Total RNA was extracted from NcCK, Nc7T, and Nc10T samples using an RNA extraction kit (Promega, Madison, WI, USA) and served as the template for reverse transcription. To enrich for circular RNAs, the RNA templates were treated with 3 U/µg of RNase R enzyme to degrade linear RNAs. Following incubation at 37 °C for 15 min, the treated RNA was reverse transcribed using random primers. The resulting cDNA was used as the template for PCR amplification. The PCR reaction components and cycling conditions were set up according to the method described by Geng et al. [15]. The PCR products were separated by 1.5% agarose gel electrophoresis, visualized, and photographed.

### 2.11. RT-qPCR Validation

The *actin* gene (Gene ID: AB023025.1) was used as the internal reference. The RT-qPCR reaction components and cycling conditions were established following the protocol described by Geng et al. [15]. The relative expression levels of the circRNAs were calculated using the 2^−ΔΔCT^. method. Data analysis and graphing were performed using GraphPad Prism 8 software. Experimental data are presented as the mean ± SD. Statistical significance was determined using the Student’s *t*-test (ns: *p* > 0.05, *: *p* < 0.05, **: *p* < 0.01, ***: *p* < 0.001, ****: *p* < 0.0001).

## 3. Results

### 3.1. Identification and Analysis of circRNAs in V. ceranae

**Figure 1:**
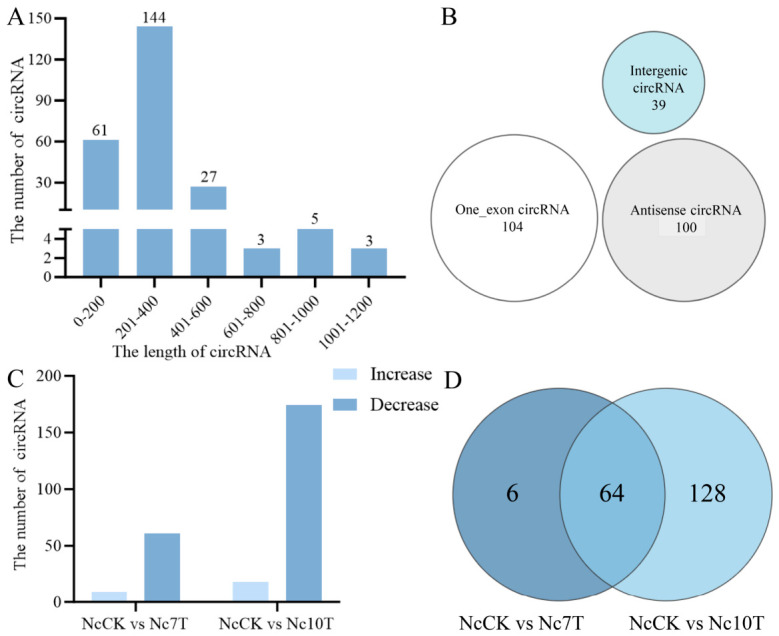
Differential analysis of circRNAs in *Vairimorpha ceranae* infecting the *Apis mellifera ligustica* workers. (**A**) Length distribution of circRNAs. (**B**) Category of circRNAs. (**C**) Number of differentially expressed circRNAs (DEcircRNAs). (**D**) Venn diagram of DEcircRNAs. NcCK: *V. ceranae* spores, Nc7T; Nc10T: *V. ceranae* in the workers’ midguts at 7 days and 10 days post inoculation with spores.

A total of 243 circRNAs were identified, with an average length of 299 bp. The majority of these circRNAs (144, 59.26%) ranged from 201 to 400 nt in length, as shown in Figure 1A. Among them, the most abundant type was single-exon circRNAs (104), followed by antisense circRNAs (100) and intergenic circRNAs (39), accounting for 42.80%, 41.15%, and 16.05%, respectively (Figure 1B).

### 3.2. Differential Expression Profile of circRNAs in V. ceranae

There were 70 and 192 differentially expressed circRNAs (DEcircRNAs) in *V. ceranae* identified from NcCK vs. Nc7T and NcCK vs. Nc10T group, respectively (Figure 1C). In the NcCK vs. Nc7T group, nine upregulated and 61 downregulated circRNAs were detected, with the length distribution among 2.67~27.00 nt. The most upregulated and downregulated ones were novel-circ-000104 and novel-circ-004707, respectively. In the NcCK vs. Nc10T group, there were 18 upregulated and 174 downregulated circRNAs, with novel-circ-000104 and novel-circ-006373 being the most upregulated and downregulated ones. In addition, Venn diagram analysis revealed that 64 DEcircRNAs were shared by these two comparison groups, while the quantities of unique ones were six and 128, respectively (Figure 1D).

**Figure 2:**
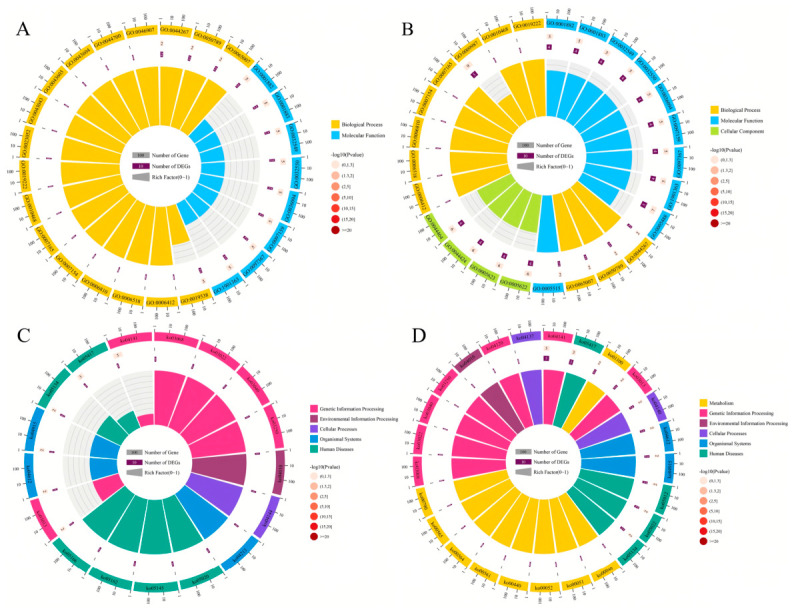
GO terms and KEGG pathways enriched by the parental genes of DEcircRNAs of *Vairimorpha ceranae* infecting the *Apis mellifera ligustica* workers. (**A**) Loop graphs of GO terms enriched by parental genes of DEcircRNAs in the midguts at 7 dpi. (**B**) Loop graphs of GO terms enriched by parental genes of DEcircRNAs in the midguts at 10 dpi. (**C**) Loop graphs of KEGG pathways enriched by parental genes of DEcircRNAs in the midguts at 7 dpi. (**D**) Loop graphs of KEGG pathways enriched by parental genes of DEcircRNAs in the midguts at 10 dpi.

The parental genes of DEcircRNAs in the NcCK vs. Nc7T comparison group were involved in 12 GO terms across the three major categories: biological process, molecular function, and cellular component. The most abundantly annotated terms included cellular process, cell, cell part, biological regulation, and metabolic process (Figure 2A). Those DEcircRNAs in the NcCK vs. Nc10T comparison group were engaged in 14 GO terms, with cellular process, metabolic process, binding, cell, and cellular component being the most represented (Figure 2B). Additionally, the parental genes of DEcircRNAs in the midgut at 7 dpi were associated with 17 pathways, including endocytosis, spliceosome, and ribosome biogenesis in eukaryotes (Figure 2C). In the midgut at 10 dpi, the parental genes of DEcircRNAs were relevant to 55 pathways, such as protein processing in endoplasmic reticulum, ribosome, and metabolic pathways (Figure 2D).

### 3.3. Analysis of the ceRNA Regulatory Network

**Figure 3:**
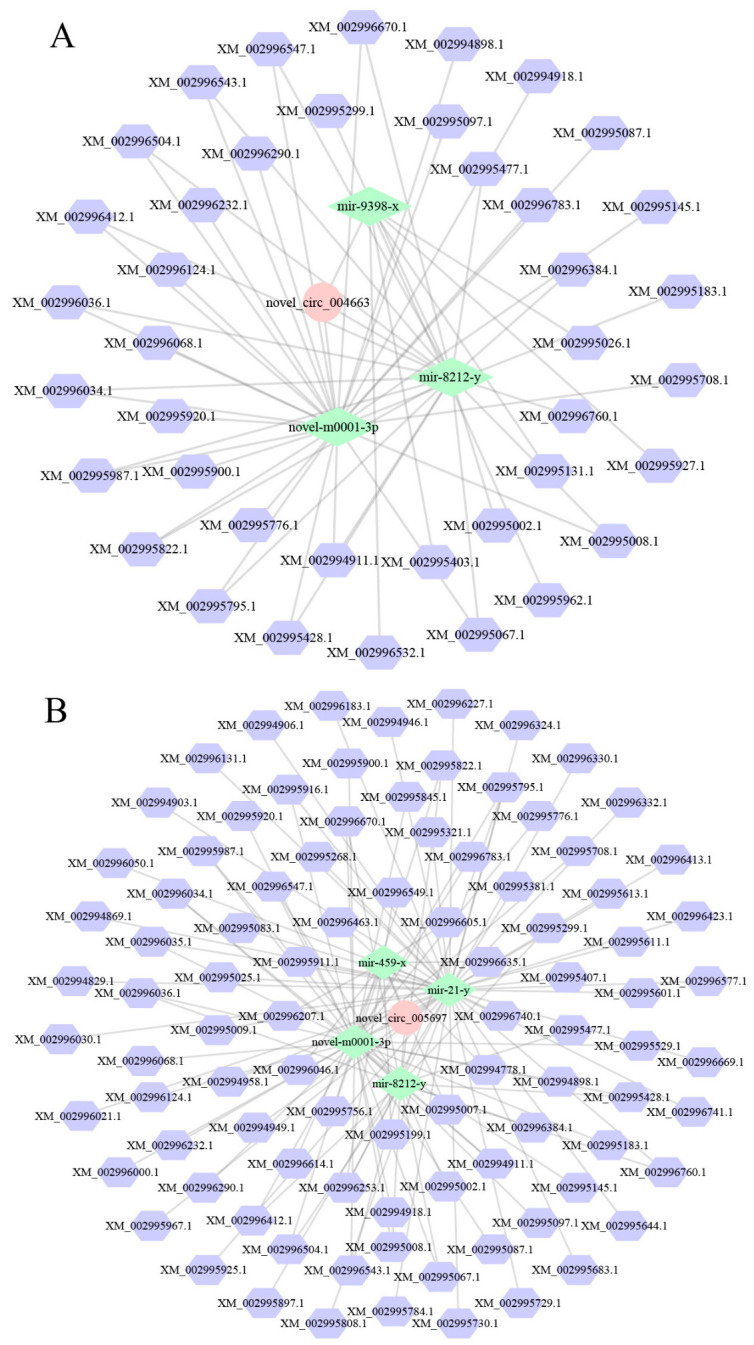
The DEcircRNA–DEmiRNA–DEmRNA regulatory network in *Vairimorpha ceranae*. (**A**) DEcircRNA–DEmiRNA–DEmRNA regulatory network at 7 dpi. (**B**) DEcircRNA–DEmiRNA–DEmRNA regulatory network at 10 dpi. Hexagons represent DEmRNAs, diamonds represent DEmiRNAs, and circles represent DEcircRNAs. DE represents differentially expressed.

In the NcCK vs. Nc7T comparison group, 32 DEcircRNAs were predicted to target 23 DEmiRNAs, which further targeted 353 mRNAs. Among these, nce-novel-m0001-3p, nce-miR-216-x, and nce-miR-8212-y respectively interacted with ten, seven, and seven DEcircRNAs, whereas novel-circ-003443 and novel-circ-004663 each bound to four DEmiRNAs, as shown in Figure 3A. Comparatively, 86 DEcircRNAs in the midgut at 10 dpi could target 32 DEmiRNAs, further targeting 515 mRNAs. Here, nce-novel-m0001-3p, nce-miR-8212-y, and nce-miR-424-x were observed to interact with 21, 20, and 11, respectively, while novel-circ-005697, novel-circ-004633, and novel-circ-004663 linked to four, three, and three DEmiRNAs, respectively (Figure 3B).

**Figure 4:**
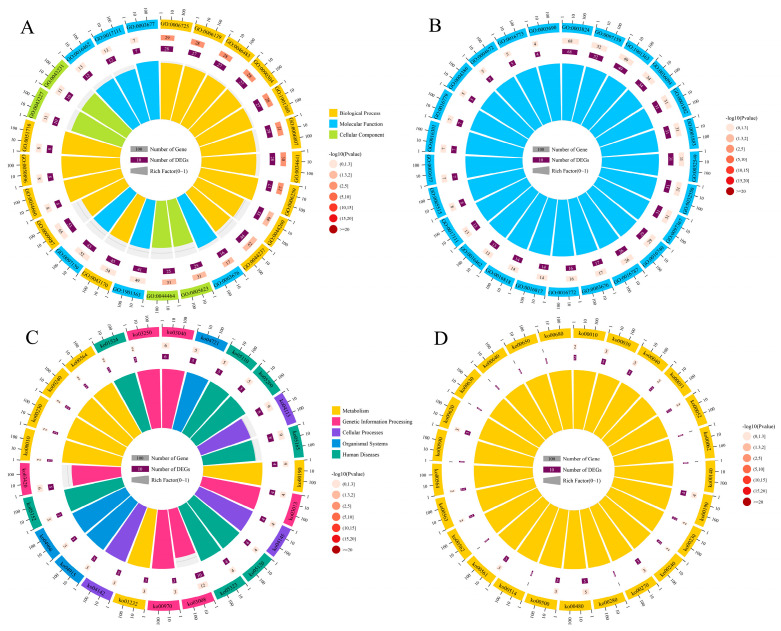
GO terms and KEGG pathways annotated by the target mRNAs in ceRNA networks in *Vairimorpha ceranae* infecting the *Apis mellifera ligustica* workers. (**A**) Loop graphs of GO terms annotated by target mRNAs in the midguts at 7 dpi. (**B**) Loop graphs of GO terms annotated by target mRNAs in the midguts at 10 dpi. (**C**) Loop graphs of KEGG pathways annotated by target mRNAs in the midguts at 7 dpi. (**D**) Loop graphs of KEGG pathways annotated by target mRNAs in the midguts at 10 dpi.

It was found that the target mRNAs in the ceRNA network in the midgut at 7 dpi could be annotated to nine functional terms related to biological process such as cellular process, metabolic process, and single-organism process, seven terms relative to cellular component, like cell, cell part, and organelle, and three terms associated with molecular function including binding, catalytic activity, and transporter activity (Figure 4A). In the midgut at 10 dpi, the target mRNAs can be annotated to nine biological process terms (e.g., metabolic process, cellular process, single-organism process), seven cellular component terms (e.g., cell, cell part, organelle), and three molecular function terms (e.g., catalytic activity, binding, transporter activity) (Figure 4B). In addition, those target mRNAs in the NcCK vs. Nc7T comparison group were annotated to 179 pathways, such as metabolic pathways, biosynthesis of secondary metabolites, and ribosome biogenesis in eukaryotes (Figure 4C), while the targets in the NcCK vs. Nc10T comparison group were annotated to 198 pathways, such as metabolic pathways, biosynthesis of secondary metabolites, and ribosome biogenesis in eukaryotes (Figure 4D).

**Figure 5:**
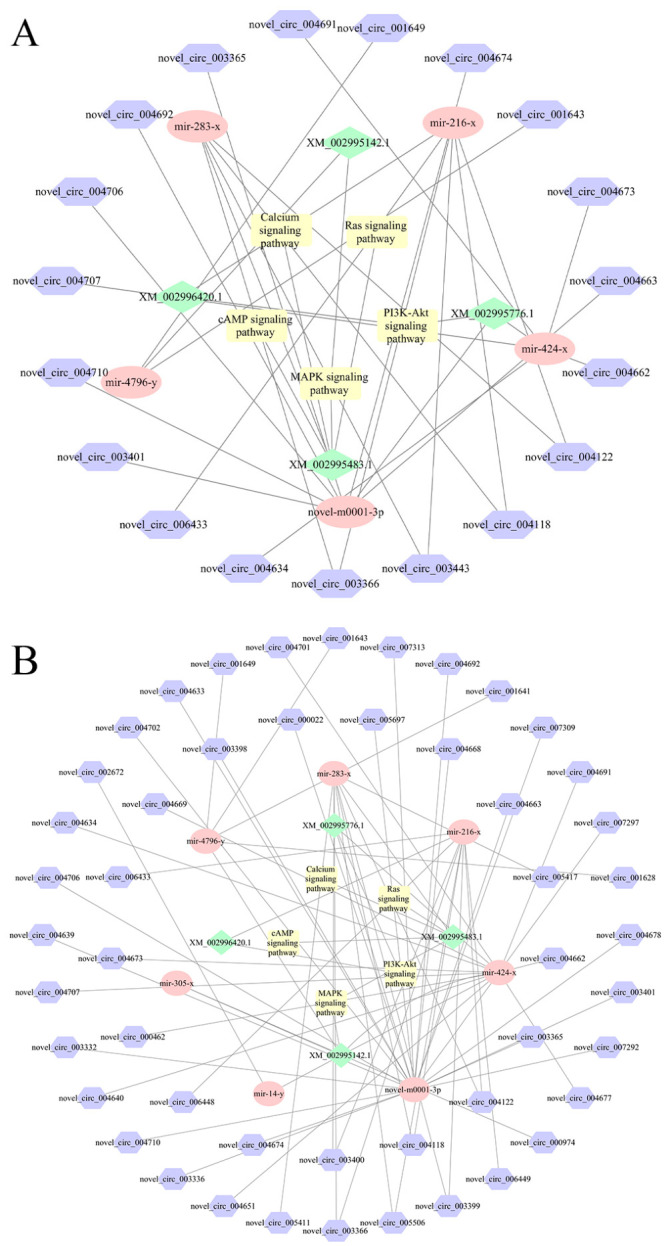
(**A**) DEcircRNA–DEmiRNA regulatory networks associated with proliferation-related signaling pathways in *Vairimorpha ceranae* at 7 dpi. (**B**) DEcircRNA–DEmiRNA regulatory networks associated with proliferation-related signaling pathways in *V. ceranae* at 10 dpi. Hexagons represent DEmRNAs, diamonds represent DEmiRNAs, circles represent proliferation-related signaling pathways, and rectangles represent DEcircRNAs.

Further analysis revealed that within the ceRNA network of the NcCK vs. Nc7T comparison group, 19 DEcircRNAs could target five DEmiRNAs, which in turn were implicated in regulating four mRNAs. These interactions were associated with five proliferation-related pathways: the Ras signaling pathway, cAMP signaling pathway, PI3K–Akt signaling pathway, MAPK signaling pathway, and calcium signaling pathway. Similarly, in the NcCK vs. Nc10T comparison group, 49 DEcircRNAs could target seven DEmiRNAs, subsequently regulating four mRNAs involved in the same five proliferation-related pathways: Ras, cAMP, PI3K–Akt, MAPK, and calcium signaling pathways (Figure 5, Table 2).

### 3.4. Investigation of the Protein-Coding Potential of DEcircRNAs

In the NcCK vs. Nc7T comparison group, a total of 87 IRES elements were identified within the DEcircRNAs, while 286 IRES elements were detected within the DEcircRNAs in the NcCK vs. Nc10T comparison group. Among these, 79 IRES elements were common to both comparison groups.

### 3.5. Validation of Back-Splicing Junctions and Expression Trends of DEcircRNAs

**Figure 6:**
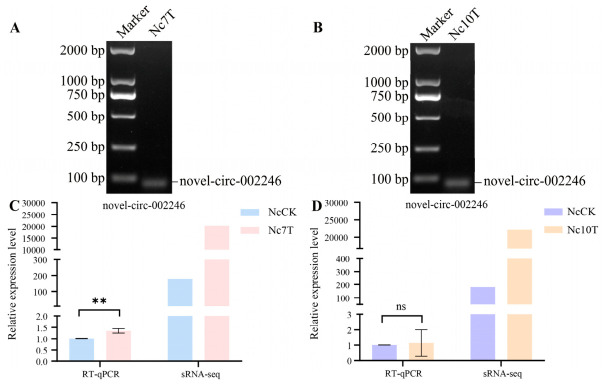
RT-PCR and RT-qPCR validation of novel-circ-002246 in *Vairimorpha ceranae*. (**A**) Agarose gel electrophoresis for PCR amplification product, lane 1: DNA marker, lane 2: PCR product of novel-circ-002246 in Nc7T. (**B**) Agarose gel electrophoresis for PCR amplification product, lane 1: DNA marker, lane 2: PCR product of novel-circ-002246 in Nc10T. (**C**) RT-qPCR result of novel-circ-002246 in *V. ceranae* infecting the midgut at 7 dpi (Nc7T). (**D**) RT-qPCR result of novel-circ-002246 in *V. ceranae* infecting the midgut at 10 dpi (Nc10T). The experimental data were presented as the mean ± SD and subjected to the Student’s *t* test; ns: *p* > 0.05; **: *p* < 0.01. NcCK: *V. ceranae* spores, Nc7T and Nc10T: *V. ceranae* in the workers’ midguts at 7 days and 10 days post-inoculation with spores.

RT-PCR with divergent primers was performed to validate novel-circ-002246 from the NcCK vs. Nc7T and NcCK vs. Nc10T comparison groups, the electrophoresis indicated that novel-circ-002246 exhibited amplification products consistent with their expected sizes (Figure 6A,B). Further RT-qPCR assays showed that novel-circ-002246 was upregulated in both the NcCK vs. Nc7T and NcCK vs. Nc10T comparison groups, consistent with the sequencing data (Figure 6C,D).

## 4. Discussion

In our previous studies, we characterized the host response to *V. ceranae* infection, specifically detailing the dynamic alterations in the host’s circRNA expression profiles [18,19]. While these studies provided valuable insights into host immunity and pathogenesis from the host’s perspective, the molecular mechanisms employed by the parasite itself remain largely unexplored. The existence of circRNAs in *V. ceranae* was initially established by Guo et al. [13], who identified 204 circRNAs exclusively from purified dormant spore samples. Whereas their pioneering work provided the first global evidence of circRNAs in Microsporidia, it was limited to the environmental survival stage of the pathogen. The dynamic expression patterns and regulatory roles of circRNAs during the active, intracellular infection process remain completely unexplored. Therefore, building upon previous findings, our current work represents comparative circRNA profiling of *V. ceranae* across the dynamic infection timeline (comparing dormant spores with *V. ceranae* in host midguts at 7 dpi and 10 dpi). By identifying DEcircRNAs, constructing functional interaction networks during infection, and exploring their protein-coding potential, this research contributes novel mechanistic insights into circRNA-mediated host–pathogen interactions.

Studies have shown that in eukaryotes, circRNAs exert diverse functions through their unique circular structure, acting as miRNA sponges, protein decoys, or transcriptional regulators [20]. Here, following transcriptome sequencing of the *V. ceranae* spore samples and *V. ceranae* inoculated *A. m. ligustica* worker midgut samples, we identified a total of 243 circRNAs in *V. ceranae* during the infection. Regarding biogenic types, exonic circRNAs were the most abundant (42.80%) (Figure 1B). This distribution, predominantly featuring exonic circRNAs, is consistent with previous findings in most eukaryotes [7], indicating their potential involvement in transcriptional or post-transcriptional gene regulation through base-pairing mechanisms [8,21]. Further analysis detected 70 and 192 differentially expressed circRNAs (DEcircRNAs) in the NcCK vs. Nc7T and NcCK vs. Nc10T comparison groups, respectively, with the majority being downregulated (Figure 1C). This suggests that exogenous circRNAs derived from *V. ceranae* may be more readily detected by the host immune system and moderate downregulation could serve as a strategy to help the pathogen achieve persistent survival within the host. Intriguingly, the consistent and highly significant upregulation of novel-circ-000104 was observed in the workers’ midguts at 7 dpi and 10 dpi, implying that this circRNA may play an essential part early in the infection process. This warrants further dissection of its specific mechanisms. In this study, it was found that the parental genes of DEcircRNAs were associated with cellular processes, metabolic processes, and binding. In addition, they were enriched in an array of pathways of importance, such as protein processing in the endoplasmic reticulum, RNA transport, and ribosome. These results demonstrate that *V. ceranae* is undergoing robust protein biosynthesis, processing, and transport to adapt to the host environment and establish infection. These DEcircRNAs likely function as regulatory molecules to maintain high metabolic activity and facilitate the synthesis and secretion of virulence factors [8,22].

CircRNAs regulate cellular functions primarily by acting as miRNA sponges, binding RNA-binding proteins (RBPs), or modulating transcription [23]. Although fungal circRNA research is emerging, studies in pathogens like *Trichosporon asahii* have shown that circRNAs can function as ceRNAs to regulate parental genes during the infection process [8]. Similarly, in *Helicobacter pylori* infection, upregulation of circPGD sponges miR-4429 to promote gastric epithelial proliferation [24]. In *Drosophila*, circATP8B (2) enhances antiviral defense via interaction with Dicer-2 [25]. Here, construction of a DEcircRNA–DEmiRNA–mRNA network revealed potential ceRNA mechanisms. Notably, several DEcircRNAs showed the coordinated regulation of key signaling pathways, e.g., six DEcircRNAs (novel-circ-003366 etc.) bound to miR-4796-y and miR-283-x to influence the MAPK signaling pathway, 17 DEcircRNAs targeted three miRNAs (novel-m0001-3p etc.) to jointly modulate the PI3K–Akt signaling pathway, and four DEcircRNAs interacted with miRNAs including miR-283-x to co-regulate the cAMP signaling pathway. These interactions suggest that circRNAs may orchestrate multiple signaling cascades during infection through miRNA-mediated networks.

Further analysis delineated a ceRNA network through which DEcircRNAs modulate multiple fungal proliferation-associated signaling pathways. In the worker’s midgut at 7 dpi, 19 DEcircRNAs targeted five DEmiRNAs to regulate four mRNAs involved in five key signaling pathways: Ras, cAMP, PI3K–Akt, MAPK, and calcium/calcineurin. A more extensive network was observed in the worker’s midgut at 10 dpi, where 49 DEcircRNAs interacted with seven DEmiRNAs to regulate four mRNAs and co-regulate the same set of pathways. Functionally, these pathways collectively govern critical cellular processes. In fungi, Ras homologous proteins (e.g., SsRAS1 and SsRAS2) have been confirmed to be essential for vegetative growth, sclerotial development, and virulence [26]. The cAMP signaling pathway plays a central role in fungal proliferation by integrating environmental signals and regulating morphological transitions, metabolic reprogramming, and the expression of virulence factors [27,28]. The PI3K/AKT signaling pathway is also involved in parasite development, infection, and survival within the hosts [29]. The MAPK pathway regulates filamentous growth, invasive hyphal development, and cell wall remodeling in fungi [30]. The calcium/calcineurin signaling pathway is involved in the yeast-to-hyphae transition of *Candida albicans*, a process that is essential for invasive growth [31]. Together, these results demonstrate that *V. ceranae* is likely to integrate the aforementioned signaling pathways to initiate morphological transitions and stress responses, thereby completing the process of proliferation and infection within the host.

Recent studies have shown that certain circRNAs possess protein-coding potential through IRES-mediated translation, producing functional peptides involved in cellular processes and stress responses [13,32]. Limkul et al. [33] found that a protein encoded by a white spot syndrome virus (WSSV) circRNA CircVP28 modulated virus–receptor interactions, resulting in the blocking of viral entry. It has been confirmed that *Bombyx mori* nucleopolyhedrovirus circRNA-000010 translates a 39-amino-acid viral small peptide (VSP39), which subsequently promotes viral replication [34]. In this current work, we identified 87 and 286 IRES elements within DEcircRNAs in the midguts at 7 dpi and 10 dpi, respectively, with 79 common ones. The results indicate that a subset of circRNAs may undergo translation during infection, highlighting a potential layer of functional regulation in host–microsporidian interactions and providing a basis for further investigation into circRNA-encoded peptides in infection contexts.

Despite the comprehensive circRNA profiling presented in this study, several methodological limitations must be explicitly acknowledged. First, due to current technical constraints regarding the genetic manipulation of *V. ceranae*, our structural and expression validations were primarily focused on a limited number of candidates. Second, the proposed circRNA–miRNA–mRNA regulatory networks are largely based on bioinformatic predictions. The direct physical binding and exact regulatory functions have yet to be empirically proven. Therefore, future studies employing rigorous functional experiments, such as dual-luciferase reporter assays, will be essential to experimentally validate these predicted interactions and fully elucidate the biological roles of microsporidian-derived circRNAs.

## 5. Conclusions

**Figure 7:**
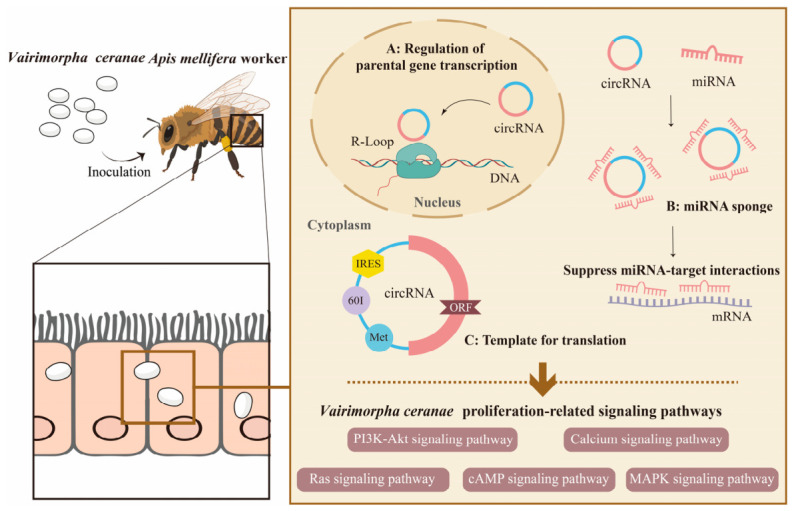
A hypothetical working model of the DEcircRNA regulated *Vairimorpha ceranae* infection of an *Apis mellifera ligustica* worker. In the right panel, the upper section illustrates the proposed molecular mechanisms of pathogen-derived circRNAs (including regulation of parental gene transcription, miRNA sponge, and template of translation), while the lower section outlines the potential biological pathways they regulate. The brown arrows indicate the hypothesized regulatory flow from the functional mechanisms to the target pathways.

In summary, this study characterizes the landscape of *V. ceranae* circRNAs, revealing that they function as key regulators of fungal proliferation and virulence by modulating critical signaling pathways (such as Ras, cAMP, and MAPK) through ceRNA networks. Furthermore, the identification of IRES elements in a subset of circRNAs indicates their potential to encode functional peptides, representing a novel layer of regulation in host–microsporidian interactions (Figure 7).

## Figures and Tables

**Table 1:** Primer sequences and relevant information in this study.

circRNA	Sequence (5′-3′)	Purpose
novel-circ-002246-F	ATGATCCAAATTTTGGTA	PCR and RT-qPCR
novel-circ-002246-R	GATGATATGGCGGCCTTC	PCR and RT-qPCR
Ncactin-F	ACAATGGTTCAGGTATCGTA	PCR and RT-qPCR
Ncactin-R	GTGCCTCATCTCCTACATAA	PCR and RT-qPCR

**Table 2:** Proliferation-related signaling pathways annotated by DEmRNAs from the ceRNA network in *Vairimorpha ceranae*.

Pathway Name	Pathway ID	mRNA Number	
		NcCK vs. Nc7T	NcCK vs. Nc10T
MAPK signaling pathway	ko04010	2	2
PI3K–Akt signaling pathway	ko04151	2	2
Ras signaling pathway	ko04014	1	1
cAMP signaling pathway	ko04024	1	1
Calcium signaling pathway	ko04020	1	1

## Data Availability

The data presented in this study are openly available in Fujian Agriculture and Forestry University at https://www.ncbi.nlm.nih.gov/bioproject/PRJNA562784 (accessed on 25 February 2026), reference number PRJNA562784.
